# Metabolic syndrome among 13 year old adolescents: prevalence and risk factors

**DOI:** 10.1186/1471-2458-14-S3-S7

**Published:** 2014-11-24

**Authors:** AA Fadzlina, Fatimah Harun, MY Nurul Haniza, Nabilla Al Sadat, Liam Murray, Marie M Cantwell, Tin Tin Su, Hazreen Abdul Majid, Muhammad Yazid Jalaludin

**Affiliations:** 1 Department of Paediatrics, Faculty of Medicine, University Malaya, Malaysia; 2 Centre for Population Health (CePH), Department of Social and Preventive Medicine, Faculty of Medicine, University Malaya, Malaysia; 3 Centre for Public Health, Queen's University of Belfast, Belfast, Ireland

**Keywords:** obesity, metabolic syndrome, adolescents, sleep duration

## Abstract

**Background:**

Obesity and metabolic syndrome is prevalent among Malaysian adolescents and has been associated with certain behavioural factors such as duration of sleep, screen time and physical activity. The aim of the study is to report the prevalence of overweight/obesity, metabolic syndrome and its risk factors among adolescents.

**Methods:**

A multi-staged cluster sampling method was used to select participants from urban and rural schools in Selangor, Perak and Wilayah Persekutuan Kuala Lumpur. Participants underwent anthropometric measurement and physical examination including blood pressure measurement. Blood samples were taken for fasting glucose and lipids and participants answered a self-administered questionnaire. Overweight and obesity was defined using the extrapolated adult body mass index (BMI) cut-offs of >25 kg/m^2 ^and >30 kg/m^2^, according to the International Obesity Task Force (IOTF) criteria. Metabolic syndrome was defined based on International Diabetes Federation (IDF) 2007 criteria.

**Results:**

Data were collected from 1361 participants. After excluding incomplete data and missing values for the variables, we analysed a sample of 1014 participants. Prevalence of overweight and obesity in this population was 25.4% (N = 258). The prevalence of metabolic syndrome was 2.6% in the population and 10% among the overweight and obese adolescents. Participants who slept between 7 and 9 hours a day has a lower risk of developing metabolic syndrome OR 0.38(0.15-0.94).

**Conclusion:**

Our results provide the prevalence of metabolic syndrome in Malaysian adolescents. Adequate sleep between 7 and 9 hours per day reduces the risk of developing metabolic syndrome.

## Introduction

The International Obesity Task Force (IOTF) in the year 2000, established the definition of overweight and obesity in childhood, based on pooled international data for body mass index (BMI) linked to the adult overweight and obesity cut-off of 25 kg/m^2 ^and 30 kg/m^2 ^respectively [1]. Over the recent years, the prevalence of childhood obesity has increased rapidly worldwide [2]. In Malaysia, the prevalence of overweight and obesity in children and adults has followed a similar trend [3-5]. With the growing obesity epidemic within the adolescent population, this has led to the increasing frequency of metabolic syndrome within this population [6,7].

Metabolic syndrome is an interrelated cluster of conditions including visceral adiposity, dyslipidaemia, hypertension and hyperglycaemia. Reaven and his colleagues [8] first described metabolic syndrome in 1988. However, prior to 2007, there was no uniform or standard criteria to diagnose metabolic syndrome in children and adolescents. A few definitions of metabolic syndrome that has been adapted for use in children and adolescents includes the National Cholesterol Education Program Adult Treatment Panel III (ATP III) [9], American Association of Clinical Endocrinologists (AACE) [10], World Health Organization (WHO) [11,12] and European Group for the Study of Insulin resistance (EGIR) [13]. Due to the differences in its definition, International Diabetes Federation (IDF) consensus was developed in 2007 to define metabolic syndrome in children and adolescents [14]. The IDF definition is divided into age groups due to the age related differences in blood pressure, lipid levels, and body size in children and adolescents [14]. As there was insufficient data available in younger children, the definition of metabolic syndrome is only applicable in children of 10 years of age and older. Metabolic syndrome was defined as the presence of, abdominal obesity and 2 or more of these criterias (elevated triglycerides, low level of high density lipoprotein-cholesterol, high blood pressure and high fasting plasma glucose). With this new definition, few studies have reported the prevalence of metabolic syndrome in their population [7,15-17].

Previous studies had shown associations between certain behavioral characteristics and obesity as well as metabolic syndrome. For example, short sleep duration has been associated with overweight, obesity and metabolic risk factor [18-20]. A study by Chen et al. has shown that adequate sleep is associated with good health status and adoption of health-related behavior [21]. Prolonged screen time [22,23] and physical inactivity [24,25] however had been identified to be inversely related to metabolic syndrome and its risk factors. These two factors may impose a major problem to the country as it has been recently found that about 64% of Malaysian adolescents are physically inactive [26].

Data regarding the prevalence of metabolic syndrome amongst Malaysian adolescents and their behavioral characteristics is still lacking. Realizing the importance of such data and the health implications, a population based study was conducted to determine the prevalence of obesity, and metabolic syndrome and its risk factors among Malaysian adolescents.

## Methodology

### Ethics statement

The study received ethical approval from the Medical Ethics Committee, University Malaya Medical Centre (IRB Refence number 896.34)

### Study design

This study was done in conjunction with the Malaysian Health and Adolescents Longitudinal Research Study (MyHeARTs), an on-going prospective longitudinal cohort study on risk factors for chronic non-communicable diseases among adolescents. The details of the study has been described previously [27].

This study was conducted in three states in the northern and central zone of Peninsular Malaysia - Perak, Selangor and Wilayah Persekutuan Kuala Lumpur. Participants comprised of both male and female students, aged 13 years old, who were in their first year of the public secondary school (Form 1) and able to read and understand the national language of Malaysia, which is Bahasa Melayu. Participants from boarding, religious and vernacular schools were excluded from the study as they were not representative of the majority of students in Malaysian schools. The total sample size calculated for this study was 1500, using the formula *n=(z^2^.p.q/r.e^2^)xDeff*, whereby *p *(prevalence) as 33% using the estimated prevalence of smoking among adolescents in Malaysia from the Global Youth Tobacco Survey (GYTS) in 2003, and *r *(response rate) of 50% based from the MyHeARTs feasibility study conducted in October to November 2011. As our study is mainly looking at prevalence of metabolic syndrome in the population, the sample size needed for our study using the above formula, is 330 based on the metabolic syndrome prevalence (4.6%) in our neighbouring country [24].

The sampling of the study was done using a multi-staged cluster sampling design. A complete list of secondary schools in Perak (238 schools), Selangor (261 schools) and Wilayah Persekutuan Kuala Lumpur (96 schools) was obtained from the Department of Education of Malaysia, and the list was used as a sampling frame. 15 schools were randomly selected as a cluster from the computer-generated random number lists to ensure randomization. Eight schools were from urban area whereas 7 schools were from rural area. Subsequently, all Form 1 students (age 13 years old) who fulfilled inclusion criteria were invited to participate in the study. Consent form for parents, ascent agreement for participants and detailed infromation sheets about the study were distributed to them. Participants were students who attended school on the day of the data collection and handed in the agreed consent form and ascent forms. Students without parental consent and individual agreement were excluded from the study. All participations were voluntary.

### Questionnaire, anthropometic measurement and physical examination

Participants were asked to fill a standardized pre-tested questionnaire under the supervision of trained enumerators. The questionnaire collected information on the participants socio-demographic details such as date of birth, age, gender and ethnic group and daily activities such as duration of sleep and screen time. To minimize the variability in measurements, the research team members attended a special workshop where appropriate training was provided for standardization of measurement. Body weight was measured in light clothing without shoes and socks using a calibrated digital electronic weighing scale (Seca 813, Seca, UK) recorded to the nearest 0.1 kilogram. Height was measured using a calibrated vertical stadiometer (Seca Portable 217 Seca, UK) recorded to the nearest 0.1 centimetre. Body mass index (BMI) was calculated using weight (kg) divided by the square of height (m^2^) and expressed as kg/m^2^. Waist circumference was measured using ergonomic circumference measuring tape (Seca 201, Seca, UK), measured at the midpoint between the lowest rib margin and the iliac crest and recorded in the nearest 0.1 centimetre. Blood pressure was measured using mercurial sphygmomanometer (Spirit CK-101C, Spirit Medical Co., Taiwan) and stethoscope after a 5 minutes rest, in a seated position with the right upper arm positioned at the heart level and the feet flat on the ground [28]. Body fat percentage was measured using a portable Body Composition Analyzer (Tanita SC-240, Tanita, Japan) and expressed by percentage (%).

### Biochemical analysis

Venous blood samples were taken after an overnight fast for at least ten hours prior to the study visit. Blood samples were collected by a trained phlebotomist and medical officers. Approximately 2 mls of blood was collected into sodium fluoride test tube for the measurement of fasting blood glucose. Another approximately 3 mls blood sample was collected in a plain test tube for the measurement of fasting lipids (triglyceride, total cholesterol, high density lipoprotein-cholesterol (HDL-C) and low density lipoprotein cholesterol (LDL-C). All blood samples were properly labelled and transported to a private certified central laboratory for analysis.

### Definitions

Overweight and obesity was defined using the IOTF criteria with extrapolation to adult BMI cut-offs of 25 kg/m^2 ^for overweight (21.91 kg/m^2 ^for males and 22.58 kg/m^2 ^for female) and 30 kg/m^2 ^for obesity (26.84 kg/m^2 ^for male and 27.76 kg/m^2 ^for female) [1]. Central obesity was defined by waist circumference ≥90th percentile according to waist circumference percentile curves for Malaysian children and adolescents [29]. The cut-off value used was 83.8 cm for male and 78.8 cm for female [29]. Metabolic syndrome and its risk factors were determined based on the International Diabetes Federation (IDF) 2007 definition of metabolic syndrome [14]. Participants were diagnosed with metabolic syndrome if they had central obesity (waist circumference ≥90th percentile) plus at least 2 of the following criteria: (1) triglycerides ≥1.7 mmol/l, (2) HDL-cholesterol <1.03 mmol/l, (3) systolic blood pressure ≥130 mmHg or diastolic blood presure ≥85 mmHg, (4) fasting plasma glucose ≥5.6 mmol/l or known type 2 diabetes mellitus [14].

### Statistical analysis

All participants' demographic information, anthropometric measurements and laboratory results were entered and analyzed using SPSS Software Version 19. Only completed data were analyzed. The comparison of socio-demographic data with IOTF BMI and metabolic syndrome were obtained using descriptive statistics. Independent t-tests were used to compare mean values of metabolic syndrome risk factors by gender. Independent t-tests were also used to compare participants' school location and risk factors for metabolic syndrome. One-way ANOVA was used to determine the difference in means between ethnic groups and each risk factors for metabolic syndrome while logistic regression was used to determine the association between sleep duration and metabolic syndrome.

## Results

Data was collected from 1361 participants who volunteered to be in the study. After excluding incomplete data and missing values for the variables, the total number of participants eligible for analysis was 1014. The socio-demographic characteristics of the participants are shown in Table 1. The majority were female, 61.8% (N = 627) while males comprised 38.2% (N = 387). Among the ethnic groups, Malays constituted the majority, 83.6% (N = 848), followed by Chinese 8.8% (N = 89), Indian 4.8% (N = 49) and other ethnic groups 2.8% (N = 28). Students from urban (53.7%, N = 545) and rural locations (46.9%, N = 463) were equally represented. The prevalence of overweight and obesity in this population was 25.4% (N = 258), whereby 16.0% (N = 162) were overweight and 9.4% (N = 96) were obese.

**Table 1:** Socio-demographic characteristics between non-obese and overweight/obese group (N = 1014)

	Non-obese (N = 756) Mean (SD) or Percentage (%)	Overweight/Obese (N = 258) Mean (SD) or percentage (%)	Total (N = 1014) Mean (SD) or percentage (%)
**Total**	74.6%	25.4%	100%
**Age**	12.87 ± 0.32	12.88 ± 0.36	12.88 ± 0.33
**Gender**			
Male Female	36.7% 63.2%	42.2% 57.8%	38.2% 61.8%
**Ethnicity**			
Malay Chinese Indian Others	83.9% 8.1% 5.0% 2.9%	82.6% 10.9% 4.3% 2.3%	83.6% 8.8% 4.8% 2.8%
**Location**			
Urban Rural	52.7% 47.2%	56.6% 43.4%	53.7% 46.3%

Each metabolic risk factor among the non-obese, and the overweight and obese group was studied and the results are shown in Table 2. The overweight and obese group showed a much higher percentage of each individual metabolic risk factors compared to the non-obese. The major difference was seen in waist circumference, 65.12% (CI 59.26-70.97) of overweight and obese group had waist circumference ≥90th percentile in comparison to 0.93% (CI 0.24-1.61) in the non-obese group.

**Table 2:** Comparison of risk factors for metabolic syndrome among non-obese and overweight/obese participants

Risk factors	Non-obese (N = 756) % (CI)	Overweight/Obese (N = 258) % (CI)
Waist circumference (≥90th percentile)	0.93 (0.24-1.61)	65.12 (59.26-70.97)
Systolic BP (≥130 mmHg)	1.19 (0.42-1.97)	13.95 (9.7-18.21)
Diastolic BP (≥85 mmHg)	3.17 (1.92-4.43)	10.07 (6.38-13.78)
Triglyceride (≥1.7 mmol/l)	3.57 (2.25-4.90)	15.50 (11.06-19.95)
HDL-cholesterol (<1.03 mmol/l)	3.04 (1.82-4.27)	15.89 (11.4-20.38)
Fasting glucose (≥5.6 mmol/l)	3.17 (1.92-4.43)	4.26 (1.78-6.75)

BP: Blood pressure, HDL-Cholesterol: High Density Lipoprotein-Cholesterol

Table 3 shows the individual risk factors of metabolic syndrome among the overweight and obese participants. Overweight and obese participants with metabolic syndrome had statistically significant higher mean waist circumference, systolic and diastolic blood pressure and triglyceride levels, and lower HDL-cholesterol levels. Fasting blood glucose was also found to be higher in the metabolic syndrome group. However, the result was not statistically significant.

**Table 3:** Risk factors of metabolic syndrome among the overweight/obese group

Risk factors	Metabolic syndrome	No metabolic syndrome	p-value
Waist circumference (cm)	93.21 ± 8.07	84.19 ± 9.21	p < 0.001
Systolic BP (mmHg)	123.31 ± 11.82	115.75 ± 11.93	p = 0.002
Diastolic BP (mmHg)	78.92 ± 9.96	72.79 ± 9.18	p = 0.002
Triglyceride (mmol/l)	2.07 ± 0.72	1.07 ± 0.44	p < 0.001
HDL-cholesterol (mmol/l)	1.09 ± 0.28	1.33 ± 0.25	p < 0.001
Fasting glucose (mmol/l)	5.31 ± 1.33	4.85 ± 0.39	p = 0.086

BP: Blood pressure, HDL-Cholesterol: High Density Lipoprotein-Cholesterol

The prevalence of metabolic syndrome in this population was 2.6%, as shown in Table 4. The prevalence was higher among males, 3.4% (n = 13) compared to females, 2.1% (n = 13). There was no difference in the prevalence of metabolic syndrome in rural versus urban areas. Among the overweight and obese participants, the prevalence of metabolic syndrome was 10.0% (n = 26). Overweight and obese males had higher percentage of metabolic syndrome, 11.9% (n = 13) compared to females, 8.7% (n = 13).

**Table 4:** Prevalence of metabolic syndrome among all participants (N = 1014) and among the overweight/obese group (N = 258)

	All participants		Overweight/Obese group	
	
	Metabolic syndrome (N = 26) % (CI)	No metabolic syndrome (N = 988) % (CI)	Metabolic syndrome (N = 26) % (CI)	No metabolic syndrome (N = 232) % (CI)
**Total**	2.6 (1.6-3.5)	97.4 (96.4-98.4)	10.0 (6.4-13.8)	90.0 (86.2-93.6)
**Gender**				
Male (N = 387) Female (N = 627)	3.4 (1.6-5.2) 2.1 (0.9-3.2)	96.6 (94.8-98.4) 97.9 (96.8-99.1)	11.9 (5.7-18.1) 8.7 (4.1-13.3)	88.1 (81.9-94.3) 91.3 (86.7-95.9)
**Ethnicity**				
Malay (N = 848) Chinese (N = 89) Indian (N = 49) Others (N = 28)	2.6 (1.5-3.7) 3.4 (-0.5-7.2) 0 3.6 (-3.8-10.9)	97.4(96.3-98.5) 96.6 (92.8-100.4) 100 96.4 (89.1-103.8)	10.3 (6.2-14.5) 10.7 (0.2-22.9) 0 16.7 (0-59.5)	89.7 (85.6-93.8) 89.3 (77.0-101.5) 100 83.3 (40.5-126.2)
**Location**				
Urban (N = 545) Rural (N = 469)	2.6 (1.2-3.9) 2.6 (1.1-3.9)	97.4 (96.1-98.8) 97.4 (96.0-98.9)	9.6 (4.8-14.4) 10.7 (4.9-16.5)	90.4 (85.6-95.2) 89.3 (83.5-95.1)

Table 5 shows that participants who slept between 7 and 9 hours a day had a lower risk of developing metabolic syndrome, OR 0.38 (0.15-0.94). This factor was found to be independently associated with metabolic syndrome after controlling for screen time and physical activity. Analysis was also carried out for the association between screen time and physical activity with metabolic syndrome but no significant associations were found (results not shown).

**Table 5:** Odds ratio for metabolic syndrome based on sleeping time

Average sleep duration/day	Unadjusted model	Intermediate model	Fully adjusted model
<7 hours	0.52(0.16-1.71)	0.52 (0.16-1.17)	0.52 (0.16-1.76)
7-9 hours	0.38(0.15-0.93)*	0.38 (0.16-0.95)*	0.38 (0.15-0.94)*
>9 hours	1.00	1.00	1.00

*p value <0.05

Unadjusted model consist of bivariate relation between sleeping duration measured with metabolic syndrome. Intermediate model consist of relation between sleeping duration with metabolic syndrome after controlling for screen time (TV time during weekdays. Fully adjusted model consist of relation between sleeping duration with metabolic syndrome after controlling screen time (TV time during weekdays) and physical activity (based on PAQc).

## Discussion

This research studied the 13 years old adolescents attending the public schools in both rural and urban areas in Malaysia. The majority of the students were of Malay ethnicity followed by Chinese and Indians reflecting the ethnic population of the country [30]. The majority of the participants in this study were females, by voluntary enrolment, although the gender of the students in each state were equally distributed [31].

This study revealed that the prevalence of overweight and obesity was high at 25.4% whereby 16.0% were overweight and 9.4% obese. A study by Ismail et al. had shown an increase in the prevalence of overweight and obesity in Malaysian children from 20.7% in 2002 to 26.5% in 2008 [3], whereas a recent study by Wee et al. showed a higher prevalence of 34.2% [5]. Compared to their Asian counterparts, the prevalence of overweight and obesity of the adolescents in this study is much higher than in the Philippines, 4.8% [32], in Thailand 7.6% overweight and 9.0% obese [33] and in Vietnam 17.8% and 3.2% overweight and obese respectively [34], as well as in China, where the prevalence of overweight and obesity combined was documented as 20% [35]. This study showed that the prevalence of overweight and obesity in Malaysian adolescents is higher compared to other South East Asian countries, mimicking the adult obesity prevalence [4].

The prevalence of metabolic syndrome amongst the 13 years old adolescents in this study was 2.6%, which is lower compared to 4.5% in the United States [7], and 4.6% in Vietnam [16], but higher compared to 1.8% in Korea [15]. Our study also showed a high prevalence of metabolic syndrome among overweight and obese adolescents (10.0%), whereby 1 in 10 overweight and obese adolescents had metabolic syndrome. However, this is lower than estimates from a community study done by Jalaludin MY et al [17] in Malaysia, and other countries in Europe, Brazil and China [36-40].

Not surprisingly, the individual risk factors of metabolic syndrome (central obesity, high triglyceride, low HDL-cholesterol, high blood pressure and high fasting blood glucose) were found to be higher in overweight and obese group in comparison to the non-obese group. Overweight and obese adolescents with metabolic syndrome were also found to have a much larger waist circumference, higher systolic and diastolic blood pressure, higher triglyceride levels and lower HDL-cholesterol levels compared to the non-metabolic syndrome group. However, there was no difference in fasting blood glucose values. Therefore, it would seem prudent to screen overweight and obese adolescents for metabolic syndrome in this population as most of them were asymptomatic for any medical illness such as type 2 diabetes mellitus or hypertension. Intervention programmes aimed at combating metabolic syndrome are necessary to reduce early cardiovascular disease and type 2 diabetes mellitus [41-43].

Previous studies has shown an association between sleeping duration [19-21,44,45], screening time [22,23] and physical activity [24,25] with obesity and other metabolic risk factors. Sleep deprivation may lead to obesity via several mechanisms; including increased sympathetic activity, increased cortisol and ghrelin levels, and decreased leptin levels. These hormonal changes will cause an increase in hunger and appetite [46,47]. Many studies have shown a U-shaped association between sleep duration and weight gain, whereby both short duration and long duration of sleep leads to weight gain [19-21,44,45]. However, the relationship between sleep duration and metabolic syndrome remains controversial. In adults, several studies have shown a relationship between both short and long sleep duration with metabolic syndrome [48,49]. Studies regarding sleep duration and metabolic syndrome in adolescents are few and have not shown any association [19,50]. This study contributes to the body of evidence that adolescents who slept between 7 and 9 hours had lower risk to develop metabolic syndrome.

A steady decline in physical activity levels has contributed to an increase in the prevalence of obesity [51]. Decreased physical activity promotes sedentary lifestyle behaviours such as television viewing and playing computer games (screen time). This also has been contributed to the fact that parents prefer their children watching television at home rather than playing outside so they are able to complete their chores and at the same time able to supervise their children [52]. Previous studies have shown a relationship between screen time and metabolic syndrome [25,53]. However, we did not find any association between screen time and metabolic syndrome in our study population. This may be due to undereporting, as screening time was assessed as time spent for television viewing but not including time spent for internet browsing and computer games.

The increase in the prevalence of metabolic syndrome poses a public health problem in our country. The development of IDF definition has provided a standard that facilitates comparisons of study results. It helps to identify patients who are at risk of developing atherosclerotic cardiovascular disease and type 2 diabetes mellitus. It also facilitates epidemiological and clinical studies, and this in turn facilitiates prevention and treatment programmes. More research regarding the consequences of metabolic syndrome in adolescents is required. However, at present, the emerging evidence shows that children with metabolic syndrome are at an increased risk of adverse events later in life such as cardiovascular disease and type 2 diabetes mellitus [54].

Routine screening among adolescents is highly recommended to prevent future complications. Many efforts have been made to promote healthy lifestyle in Malaysia to prevent obesity and metabolic syndrome [55]. In 2009, a Cochrane systematic review has identified several potentially useful strategies to treat obesity in children and adolescents [56]. Effective interventions include the incorporation of a healthy diet, reducing sedentary behavior and becoming more physically active [57,58]. The Ministry of Health, Malaysia has lauched the National Strategic Plan for Non-Communicable Disease (NSPNCD) in 2010 that focuses on 7 strategies such as prevention and promotion, as well as clinical management of non-communicable diseases [59]. Some intervention programs have been successful, while others have shown moderate success [60,61].

Environment plays an important role in contributing to overweight and obesity problems [55]. For example, food prepared in schools are more energy dense and the snacks provided are mostly fried. This may explain why the prevalence of metabolic syndrome between urban and rural areas are similar, despite the less number of fast foods outlets in the rural areas. Healthier food options should be made available and this needs to be reinforced by the government. Regular physical activity has beneficial effects on metabolic syndrome as it has been shown that physical activity is associated with better insulin sensitivity [62] and a reduction in diastolic blood pressure [63].

As described above, many studies have shown that obesity is linked to physical inactivity, sleeping pattern and sedentary lifestyle behaviours. Hence, these are the factors that should be targeted by any intervention programs for children and adolescents.

## Competing interests

The authors declared no competing interests.

## Authors' contributions

All authors contribute to study design, revising and improving the manuscript; NAS is the principal investigator for MyHEARTS; FAA, MYJ, HAM, TTS and NAS were involved in field work and data collection; FAA, NHMY, TTS and MYJ participated in analysis and interpretation of data; FAA, FH and MYJ drafted the manuscript.
